# Macroautophagy in Endogenous Processing of Self- and Pathogen-Derived Antigens for MHC Class II Presentation

**DOI:** 10.3389/fimmu.2015.00459

**Published:** 2015-09-22

**Authors:** Fernanda V. Duraes, Jennifer Niven, Juan Dubrot, Stéphanie Hugues, Monique Gannagé

**Affiliations:** ^1^Department of Pathology and Immunology, School of Medicine, University of Geneva, Geneva, Switzerland; ^2^Division of Rheumatology, Department of Internal Medicine, University Hospital Geneva, Geneva, Switzerland

**Keywords:** macroautophagy, antigen presentation/processing, MHC class II, tolerance mechanisms, CD4-positive T-lymphocytes

## Abstract

Although autophagy is a process that has been studied for several years its link with antigen presentation and T cell immunity has only recently emerged. Autophagy, which means “self-eating,” is important to maintain cell homeostasis and refers to a collection of mechanisms that delivers intracellular material for degradation into lysosomes. Among them, macroautophagy pathway has many implications in different biological processes, including innate and adaptive immunity. In particular, macroautophagy can provide a substantial source of intracellular antigens for loading onto MHC class II molecules using the alternative MHC class II pathway. Through autophagosomes, endogenous self-antigens as well as antigens derived from intracellular pathogens can be delivered to MHC class II compartment and presented to CD4^+^ T cells. The pathway will, therefore, impact both peripheral T cell tolerance and the pathogen specific immune response. This review will describe the contribution of autophagy to intracellular presentation of endogenous self- or pathogen-derived antigens via MHC class II and its consequences on CD4^+^ T cell responses.

## Introduction

A regulated balance between biosynthesis and degradation of different cellular components is required to maintain cell homeostasis. In eukaryotic cells, two main protein degradation systems co-exist: the ubiquitin–proteasome and the lysosome (1). Classically, the proteasome degrades soluble short-lived proteins in a large cytosolic proteolytic complex, whereas long-lived proteins and organelles are degraded in vesicles by lysosomal enzymes via autophagy.

More than five decades ago, autophagosomes were observed in isolated rat liver cells. Indeed, membrane vesicles containing semi-digested mitochondria and other cytoplasmic components were visualized using electron microscopy (2) and were shown to contain lysosomal enzymes (3). These observations came soon after the discovery of a new organelle with lytic function, named the lysosome (4). In 1963, de Duve created the term *autophagy* to describe the presence of double membrane vesicles containing cytoplasmic organelles in various degrees of disintegration.

Today, autophagy [from Greek: *auto* (self), *phagos* (to eat), meaning “self-eating”] refers to the breakdown mechanism that enables cells to recycle cytoplasmic constituents by degrading defective organelles and long-lived proteins in lysosomes. Initially considered to be an important alternative energy source in response to starvation, autophagy has now been implicated in multiple biological processes, including development, aging, and regeneration (5). Aberrant regulation of autophagy induces cancer, neurodegenerative diseases, and many other disorders (6). Autophagy also has diverse functions in innate immunity: pathogen recognition, elimination of microorganisms, control of inflammation, and secretion of immune mediators (7). In addition, autophagy contributes to adaptive immunity through diverse mechanisms: endogenous antigen presentation via MHC class II molecules (8, 9) control of B and T cell function, and control of thymic T cell selection (7).

Currently, three different pathways of autophagy have been described: macroautophagy, microautophagy, and chaperone-mediated autophagy (CMA) (10). They differ mainly on the molecular pathway the products (cargo) are delivered into lysosomes.

**Figure 1:**
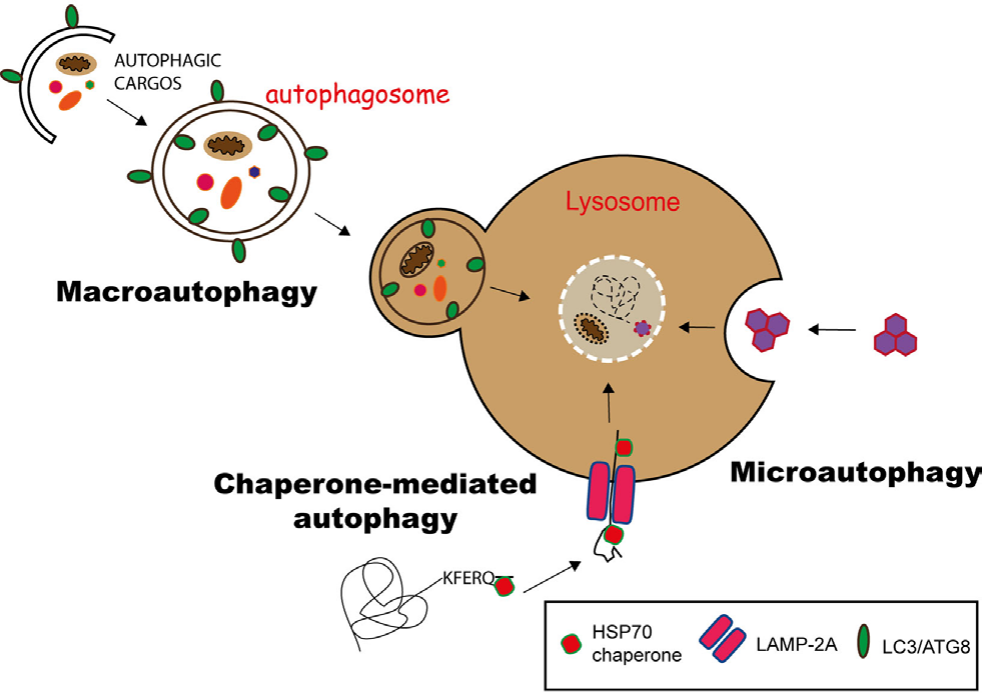
**Pathways of autophagy**. Autophagy can deliver cytosolic components to lysosomes for degradation via three different pathways. In chaperone-mediated autophagy (CMA), proteins having a KFERQ-like motif are translocated into the lysosome via the LAMP-2A transporter, with the help of Hsp70 chaperones. Microautophagy involves the sequestration of substrates via the invagination of the lysosomal membrane, while in macroautophagy, the substrates are engulfed in a double membrane vesicle, called autophagosome, which subsequently fuses with the lysosome to deliver its content for degradation.

Substrates of CMA carry a KFERQ-like signal peptide and are recognized by the chaperone HSC70 (heat shock cognate 70), forming a substrate/chaperone complex. This complex is imported into the lysosome via LAMP2a (lysosome-associated membrane protein 2a) transporter, assisted by another HSC70 member in the lysosomal lumen. This is a unique selective pathway for the delivery of proteins into lysosomes (11, 12) (Figure 1).

During microautophagy, cytoplasmic components directly gain access to the lysosome lumen via invagination and budding of its membrane. The cargo is enclosed through the formation of autophagic bodies, which are then degraded by lysosomal hydrolysis (13) (Figure 1).

Macroautophagy is the best-characterized route for lysosomal degradation of cytoplasmic constituents. During this process, cytoplasmic contents or organelles are delivered to lysosomes for degradation. The hallmark of macroautophagy is the *de novo* formation of a cytosolic double membrane vesicle. Different membrane sources can contribute to the formation of the autophagosomal membrane, including the plasma membrane, the endoplasmic reticulum (ER), and the outer mitochondrial membrane (14). The autophagosome will then fuse with late endosomes and lysosomes to deliver its contents for enzymatic degradation. The resulting macromolecules are recycled back into the cytosol, where they can be reused for anabolic or catabolic reactions (15, 16) (Figure 1).

Autophagosome formation is a complex multi-step event that is controlled by different autophagy-related genes (ATGs). At least 30 ATGs contribute to autophagy in yeast and are highly conserved among eukaryotes (17). Initial nucleation and assembly of the phagophore membrane (isolation membrane in mammals) require the action of the class III phosphatidylinositol 3-kinase (PtdIns3K) complex, which recruits multiple Atg proteins. In this process, the ubiquitin-like conjugation system Atg12–Atg5–Atg16 and Atg8 (known as LC3 in mammals) regulates autophagosome membrane elongation (18). Upon completion, all Atgs from the outer membrane are recycled. Importantly, Atg8, which is incorporated into both the inner and outer membrane of the forming autophagosome, remains associated in the inner membrane after fusion with lysosomes. Given its unique association with autophagosomes and autolysosomes, Atg8 is widely used as a marker of autophagosome formation and autophagy induction (19).

Autophagy has been described to substantially impact several aspects of innate and adaptive immunity (20). Autophagy has an intrinsic role in different cell types of the adaptive immune system. Autophagy abrogation in B cells (21), T cells (22–24), and NKT cells (25) results in decreased differentiation, effector function, and maturation. In parallel, Atg16 deficient dendritic cells (DCs) exhibit a more activated phenotype, including overexpression of co-stimulatory molecules and increased NF-kappaB activation (26). In addition to this cell intrinsic role, autophagy can impact different aspects of the adaptive immune response through its direct or indirect role in antigen presentation. Indeed autophagy can, for example, indirectly contribute to antigen presentation through its implication in the activation of various pattern-recognition receptors (PRRs) and damage-associated molecular patterns (DAMPs). In parallel, the pathway can ­control the secretion of different cytokines, mainly IL-1 beta, and therefore, contribute to the amplification or skewing of the T cell. The direct role of autophagy in antigen presentation has been described either in the donor cells or in the professional antigen-presenting cells (APCs).

This review will focus on the direct role of autophagy in APCs and its implication in delivering endogenous self- and pathogen-derived ligands for presentation via major histocompatibility complex (MHC) class II molecules. In this review, we will not discuss the implication of unusual pathways of autophagy to antigen processing. Indeed, a mini-review of the same Frontiers topic specially focuses on that point (27). Rather, we will discuss the implication of macroautophagy in MHCII-mediated antigen presentation of intracellular proteins and its effects on peripheral CD4^+^ T cell responses in inflammatory and infectious diseases.

## Autophagy in the Immune System: Endogenous MHC Class II Antigen Processing and Presentation

### MHC class I and class II classical antigen processing pathways

Antigen presentation refers to pathways involved in the effective delivery of antigens to MHC molecules. Relatively small peptides (8–10) or (15–20) amino acids are generated by proteolytic cleavage of protein substrates and displayed in the peptide-binding groove of surface expressed MHC class I or class II molecules, respectively. T cells, with their specific T-cell receptor (TCR), scan for the presence of cognate peptide–MHC complexes displayed at the cell surface of APCs. Recognition of antigenic fragments by CD4^+^ or CD8^+^ T cells is crucial to T cell activation and effector function (28).

**Figure 2:**
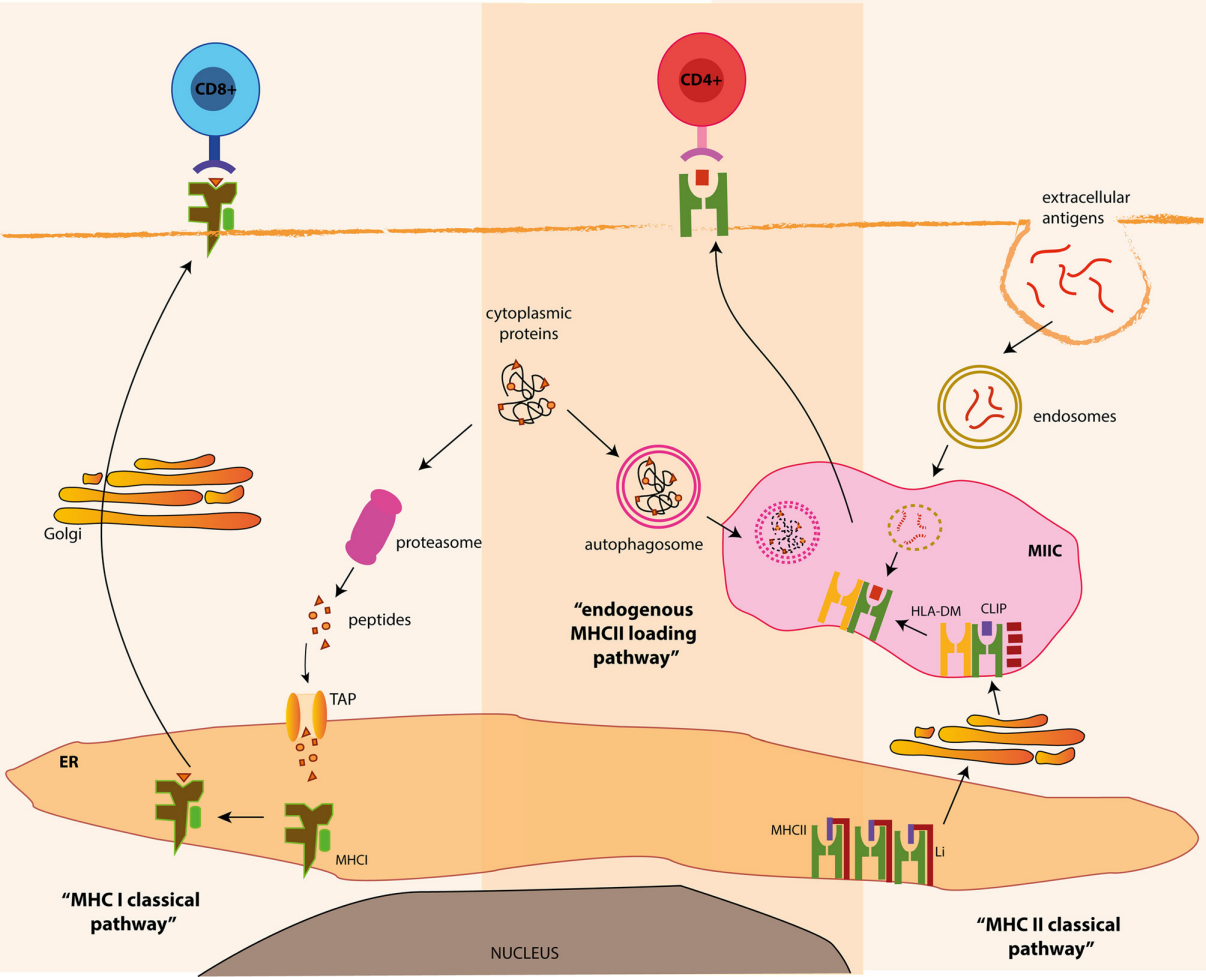
**MHC class I and class II processing pathways and autophagy**. Classically, MHC class I bound antigens are originated from intracellular proteins through proteasomal proteolysis and are transferred to the outer membrane, where the resulting peptides are presented to CD8^+^ T cells. On the other hand, MHC class II products originate from extracellular antigens, which are endocytosed and delivered to MHC class II containing compartments (MIIC), where they meet newly generated MHC class II molecules. Alternatively, autophagy can deliver cytosolic antigens for MHC class II presentation, via the fusion of autophagosomes and MIIC, for the presentation of antigens to CD4^+^ T cells.

Classically, MHC class I bound peptides are generated in the cytosol from various intracellular sources, such as cytosolic or nuclear self-proteins, proteins from intracellular pathogens or endogenous tumor antigens (29). Ubiquitinylation often targets these antigens for proteasomal degradation (30). Proteasomal products are then imported into the lumen of the ER by the transporter associated with antigen processing (TAP) (31), where they are loaded on MHC class I heterodimers. Within the ER, peptide binding is required for the correct folding of MHC class I molecules and its release from the ER. Stable peptide–MHCI complexes are exported to the cell surface via the golgi apparatus for presentation to CD8^+^ T cells (Figure 2).

In contrast, MHC class II bound epitopes classically originate from extracellular antigens (derived from foreign- or self-origin) phagocytosed by APCs and degraded by lysosomal proteolysis. These antigenic fragments are loaded onto MHC class II molecules in the so-called MHC class II compartments (MIICs) or late endosomes. MHC class II molecules are synthesized in the ER and associate with a chaperone known as the invariant chain (Ii; also known as CD74). Ii prevents premature peptide loading onto MHC class II molecules in the ER and guides newly assembled MHC class II molecules to late MIIC. Ii is then degraded in MIIC by lysosomal hydrolysis leaving the class II-associated invariant chain peptide (CLIP) in the peptide-binding groove. CLIP is replaced by high-affinity peptides with the help of the non-classical MHC class II molecule HLA-DM. Following peptide loading, peptide–MHC class II complexes are delivered to the cell surface for CD4^+^ T cell presentation (32) (Figure 2).

According to this classical view, MHC class I and class II molecules are specialized in presenting peptides derived from different origins. Through this division of labor, cytotoxic CD8^+^ or CD4^+^ helper T cells monitor the intracellular and the extracellular niches, respectively, for the presence of pathogens or for the maintenance of peripheral tolerance. However, this segregated origin of peptides can be bypassed by unconventional pathways (33). For instance, “cross-presentation” is a pathway allowing DCs to present extracellular antigens through MHC class I molecules (34, 35). Consequently, cross-presentation is an important pathway for the initiation of anti-viral cytotoxic CD8^+^ T cell responses and for the maintenance of CD8^+^ T cell tolerance (36, 37). Similarly, peptides of intracellular origin can be loaded onto MHC class II molecules.

Indeed, sequencing of peptides eluted from MHC class II molecules revealed that 20–30% of natural MHC class II ligands originate from intracellular cytosolic and nuclear proteins (38–40). These ligands can be generated either after cleavage by the proteasomal machinery (41) or via a group of processes, including CMA (reviewed elsewhere in this topic) and macroautophagy (Figure 2). In agreement, characterization of the MHC class II peptide repertoire expressed at the cell surface either under steady-state or after starvation-induced autophagy suggests that autophagy might influence CD4^+^ T cell-mediated responses to intracellular antigenic sources (42).

### Endogenous processing of intracellular antigens via autophagy for MHC class II presentation to CD4^+^ T cells: Model antigens

Pharmacological inhibitors provided the first evidence of the involvement of autophagy in endogenous MHC class II presentation to CD4^+^ T cells. Stockinger’s group compared the antigen presentation capacity of different cells transfected with C5 protein (fifth component of mouse complement). They found that B cells and fibroblasts were able to present epitopes derived from the intracellular C5 protein to CD4^+^ T cells. Interestingly, in the presence of a non-specific inhibitor of autophagy, 3-MA (3-methyl adenine) – known to inactivate class III PI3 kinase) – MHC class II presentation of endogenous C5 was abrogated (43).

Subsequent studies took advantage of the same inhibitory mechanism to show that autophagy was involved in the presentation of epitopes derived from cytosolic antigens. Transfection of a model antigen, the neomycin phosphotransferase II (NeoR) into two different cell lines, showed that MHC class II-dependent presentation of NeoR was abrogated by 3-MA inhibition, and therefore, likely to be mediated via autophagy. In parallel, upon 3-MA treatment antigen degradation was inhibited (44). In another study, using DCs transfected with *in vitro*-transcribed RNA coding for a tumor-associated cytoplasmic antigen (MUC1), the authors demonstrated that the presentation of MUC1 on MHC class II molecules required lysosomal/endosomal processing (45). Furthermore, antigen presentation of MUC1 to CD4^+^ T cells was abrogated in the presence of 3-MA, suggesting an involvement of autophagy in MUC1 processing and delivery to class II compartment.

More recently, autophagy has been shown to play a role in the presentation of citrullinated peptides from the hen-egg-white lysozyme (HEL) to CD4^+^ T cells (46). This model antigen was overexpressed at the membrane of APCs resulting in strong presentation of an immune-dominant CD4 epitope (47). Blocking autophagy in DCs, using either 3-MA treatment or Atg5 siRNA silencing, specifically inhibited the presentation of citrullinated but not native HEL peptides. In parallel, presentation of HEL-citrullinated peptides by B cells required the engagement of the B cell receptor, which was also inhibited by 3-MA treatment (46). As the presentation of citrullinated proteins plays a key role in pathogenesis of rheumatoid arthritis (48), such findings highlight the potential contribution of autophagy to the pathogenesis of a common autoimmune disease. Nevertheless, the physiological relevance of this finding needs to be expanded to more relevant autoantigens in rheumatoid arthritis.

The limitation of these studies is that they relied on artificial overexpression of model antigens, and therefore, they can only suggest an implication of autophagy pathway in the endogenous MHC class II antigen processing of physiologically expressed proteins. In addition, another major drawback, which may impede a proper assessment of how autophagy influences physiological CD4^+^ T cell responses, is the use of the pharmacological inhibitor 3-MA, which not only blocks autophagy but also affects additional biological processes (19).

The generation of labeled markers for autophagosome formation provided a better demonstration of how autophagy is involved in MHC class II presentation. To further support a broader relevance of autophagy under basal normal conditions and not only under starvation, Schimd et al. showed that low constitutive autophagosome formation occurred in a variety of human APCs, such as DCs, macrophages, and B cells (9). In this study, autophagosome formation was monitored by the accumulation of Atg8/LC3 into vesicles upon treatment with chloroquine, a blocking agent of lysosomal proteolysis. Since LC3 (the human ortholog for ATG8 in yeast) is specifically incorporated into the autophagosomal membrane upon its formation, LC3 turnover can, therefore, be used to measure autophagic activity. Autophagosomes were shown to fuse with MIIC, as evidenced by immunofluorescence co-localization of LC3-GFP, MHC class II, and HLA-DM, in both DCs and human epithelial cell lines. Importantly, silencing of Atg12 inhibited autophagosome formation and fusion with MIIC (9). In addition, a proof of concept experiment demonstrated that autophagosomes could efficiently deliver antigens to MIIC. Influenza viral protein MP1 was expressed in a fusion construct by coupling Atg8/LC3 to the C-terminus of MP1. This strategy efficiently targeted MP1 to autophagosomes and significantly enhanced its antigen presentation to CD4^+^ T cell-specific clones (9).

### Endogenous MHC class II processing of pathogen-derived antigens via autophagy

The main contribution of autophagy to antigen processing of endogenous proteins and their delivery to MIIC has been described in the context of viral or bacterial infection. Indeed, autophagy is required for efficient presentation of endogenous pathogen-derived antigens on MHC class II molecules to enhance specific CD4^+^ T cell activation.

The first viral antigen shown to be delivered to MIIC by autophagy was the Epstein–Barr virus (EBV) nuclear antigen 1 (EBNA-1) (8). In this study, the authors used EBV-transformed lymphoblastoid cells (LCLs) and EBNA-1-specific CD4^+^ T cell clones. Immunofluorescence analysis of LCLs showed that upon inhibition of lysosomal acidification, and therefore, autophagosome maturation, EBNA-1 could accumulate in cytoplasmic vesicles, which expressed the lysosomal marker LAMP1. In parallel, EBNA-1 was visualized in autophagosomes by electron microscopy. Furthermore, blocking autophagy, by treatment with 3-MA or by siRNA-mediated silencing of Atg12, resulted in reduced MHC class II-restricted CD4^+^ T cell recognition of EBNA1 (8). In the same line of this pioneer study, Leung et al. have shown that autophagy can play a role in the processing of specific CD4^+^ T cell epitopes of the EBNA-1 antigen along with other endogenous pathways (49). Interestingly, the location of native EBNA-1 within the nucleus leads to less processing and presentation on MIIC, due to the absence of autophagy within the nucleus. Indeed by mutating the nuclear localization signal of EBNA-1, the range of CD4^+^ T cell epitopes processed through autophagy was broader since the protein was more accessible for cytoplasmic autophagic degradation (49).

Another pathogen-derived antigen processed through autophagy is the immunodominant Ag85B antigen, from *Mycobacterium tuberculosis* (Mtb) (50). Mtb, amongst other pathogens, can survive in phagosomes, as part of an evasion mechanism to avoid degradation. In this context, stimulation of phagosomal maturation and lysosomal degradation via the induction of autophagy enhances Mtb clearance (51, 52), and may be required for optimal immune responses against Mtb. Indeed, *in vivo*, activation of autophagy in DCs significantly increased the presentation of Ag85B to specific CD4^+^ T cells. Mice vaccinated with Mtb-infected and rapamycin-treated DCs, exhibit a stronger specific CD4^+^ T cell response after Mtb challenge. In parallel, blocking autophagy in DCs, prior to vaccination, leads to a reduced Mtb-specific CD4^+^ T cell response (50).

A further *in vivo* study focusing on the role of autophagy during respiratory syncytical virus (RSV) infection in mice has also shown that autophagy plays a role in anti-viral CD4^+^ T cell responses. Mice having a defect in Beclin-1 (Beclin-1^+/−^), thus resulting in reduced autophagosome formation, exhibit exacerbated lung inflammation upon RSV infection, with increased Th2 responses and decreased IL-17 and IFN-γ responses. Furthermore, *in vitro* analysis of pulmonary DC from Beclin-1^+/−^ mice showed a reduction in MHC II level and co-stimulatory molecule expression. Finally, adoptive transfer of RSV-infected Beclin-1^+/−^ DC into wild type mice prior to virus challenge confirmed that the absence of autophagy within DCs leads to reduced Th1 responses and increased lung pathology (53). Recently, the same authors further dissect the contribution of autophagy in initiating and maintaining aberrant Th17 responses during RSV infection. Using mice deficient in the autophagy-associated protein, Map1-LC3b (LC3b^−/−^), they observed increased Th17 cells in lungs upon infection. In addition, airway epithelium appeared to be the primary source of IL-1β during RSV infection, whereas blockade of IL-1 receptor signaling in infected LC3b^−/−^ mice abolished IL-17-dependent lung pathology (54). Such findings highlight the role of autophagy for antigen presentation of RSV and how it can shape the adaptive anti-viral immune response.

Autophagy is also involved in antigen presentation of proteins derived from extracellular pathogens, such as the bacterium *Yersinia*. Through the type III secretion system, *Yersinia* utilizes carrier proteins, the *Yersinia* outer proteins (Yop) for the delivery of bacterial proteins into the cytosol of host cells. Interestingly by constructing a fusion antigen with the cytoplasmic translocated YopE protein, Russman et al. could demonstrate that chimeric fusion proteins are processed by autophagy, in macrophages, and presented via MHC class II to induce CD4^+^ T cell activation (55). Nevertheless, the relevance of this mechanism for Yersinia epitopes was not demonstrated.

Together, these studies suggest that autophagy induction in DCs and macrophages can enhance antigen presentation of MHC class II epitopes from intracellular pathogens in order to induce efficient CD4^+^ T cell responses. However, this scenario might not happen in all instances. Indeed, despite the fact that influenza A virus manipulates autophagy, no significant contribution of this pathway to the anti-viral CD4^+^ T cell response was demonstrated (56).

In parallel, many bacteria and viruses have developed escape mechanisms to inhibit autophagy, resulting in increased intracellular pathogen load (57–59). Whether this will negatively influence pathogenic CD4^+^ T cell responses remains to be further investigated.

### Autophagy in positive and negative selection of T cell repertoire

Autophagy plays a major role in thymic selection of a diverse T cell repertoire, and therefore, has important consequences for central tolerance induction (60).

During T cell development, T cell precursors undergo positive selection in the thymic cortex and negative selection in the thymic medulla. Positive selection allows the establishment of a functional and diverse T cell repertoire, whereas negative selection eliminates potentially auto-reactive T cells, in order to establish central tolerance toward self-antigens (61). Central tolerance is based on the presentation of self-peptides at the surface of thymic APCs, especially in thymic epithelial cells (TECs) and thymic DCs, either via MHC class I or MHC class II molecules for CD8^+^ or CD4^+^ T cell development, respectively.

The generation of a functional and self-tolerant CD4^+^ T-cell repertoire relies on the availability of a full range of self-peptides displayed by thymic APCs. The peptides presented should cover most of, if not all tissue antigens, which T cells might encounter in the periphery. Thymic APCs utilize different mechanisms in order to present a broad range of self-peptides.

Significant progresses have been made to clarify how TECs, which have low endocytic activity, can obtain self-peptides for MHC class II presentation and induction of a diverse CD4^+^ T cell repertoire devoid of auto-reactive cells (62). Recently, autophagy has been implicated in the unconventional MHC class II self-peptide loading and presentation in the thymus.

Indeed, TECs exhibit high constitutive autophagosome formation in a starvation-independent fashion (63). Neonatal lethality of mice lacking autophagy, such as ATG*5*^−/−^ or ATG7^−/−^ mice (64, 65), impedes the direct assessment of T-cell development in these conditions. Nevertheless, by transplanting embryonic Atg5^−/−^ thymi under the renal capsule of normal adult recipients, it was demonstrated that autophagy in thymic epithelium is essential for the establishment of a broad T-cell repertoire and for tolerance induction (63). In comparison to controls, transplanted thymi from knockout mice were smaller but exhibited normal epithelial differentiation and organization. In this setting, positive selection of some MHC class II-restricted TCR specificities was impaired in Atg5 deficient thymi. In contrast, absence of autophagy in TECs did not affect CD8 T cell repertoire (63). Importantly, self-tolerance was compromised when thymi from Atg5^−/−^ embryos were grafted in athymic nude mice. In this system, because of the complete deficiency of endogenous thymus, development of T cells completely relies on transplanted TECs. Between 4 and 6 weeks after grafting, transplanted mice with autophagy-deficient thymi exhibited clear signs of autoimmunity, such as progressive weight loss and inflammatory cell infiltrates, in different organs (63). These results should be, however, taken with caution since the experimental system could be geared toward autoimmunity due to the lymphopenic recipients.

In addition, autophagosomes were shown to co-localize with MIIC in both cTECs and mTECs (66) emphasizing the potential role of the pathway in thymic selection. However, more recently, the importance of autophagy in TECs for T cell development and self-tolerance establishment has been re-challenged and suggests that the lack of autophagy in TECs had a minor impact on T cell repertoire development. Transgenic mice bearing a specific suppression of Atg7 or Atg5 in epithelial cells (ATG7^f/f^ K14-Cre mice) or (ATG5^f/f^ K5-Cre mice), exhibit unaltered thymic structure, a normal T cell repertoire, and no evidence of autoimmunity (67, 68). Even though endogenous autophagy was efficiently deleted in epithelial cells of both the thymic medulla and cortex, no activation of CD4^+^ T cells nor enhanced tissue inflammation or autoimmune manifestations were observed in these models.

The difference between these models and the study by Klein et al. could be explained first by the different approaches used to abrogate autophagy in the thymus. In the first study, complete autophagy-deficient thymi were transplanted, whereas autophagy was specifically deleted in epithelial cells in the second study. A second possible explanation for this difference could also be that the two studies were carried on different mouse backgrounds. Finally, lymphopenic hosts used in the Klein’s study are known to be more permissive to autoimmunity development (69, 70).

Recently, a more refined model addressing the role of autophagy in thymic epithelium for central tolerance was carried out. A model antigen was expressed associated either to the mitochondria or to the plasma membrane. Both intracellular and membrane-bound forms of the antigen were directly presented by TECs, when transgenic thymi were transplanted under the kidney capsule of MHC class II-deficient mice. Using this scenario, a role for hematopoietic APCs in negative selection was excluded. Importantly, expression of both neo-antigen forms resulted in clonal deletion of TCR specific CD4^+^ thymocytes (71). Additionally, when autophagy was abrogated using Atg5^−/−^ thymi transplanted into transgenic mice, negative selection of T cells recognizing the membrane-associated form of the protein was not affected. However, negative selection of T cells recognizing the intracellular antigen was dependent on autophagy since it was abrogated in Atg5^−/−^ mice, firmly establishing a role for autophagy in central tolerance toward some endogenously expressed intracellular antigens.

The direct implication of efficient endogenous Ag loading into MHC class II by autophagy in mTECs was further characterized. By coupling an antigen to LC3 molecules, a new elegant model was designed to directly target the antigen to autophagosomes. In addition, expression of the fusion protein was settled under the transcriptional control of the Aire promoter. Despite the fact that both mTECs and DCs express Aire, only mTECs were able to induce effective cognate CD4^+^ T cells response, in *ex vivo* cultures, in an autophagy-dependent fashion. Moreover, using the same model, clonal CD4^+^ thymocyte deletion was also observed *in vivo*. Interestingly, mice expressing a mutated version of the fusion protein, unlinked to autophagosomes, exhibited similar negative selection of CD4^+^ thymocytes. Under these conditions, indirect presentation of this particular Ag by DC compensated the impaired direct presentation by mTECs. In addition, autophagy requirements in TECs for efficient negative selection could rely on the amount and the distribution of a given antigen (71).

Finally, a recent study has also reported an important role of autophagy in TECs for T cell selection. Using Clec16a knockdown mice in the non-obese diabetic (NOD) mouse model for type 1 diabetes, the authors unexpectedly found that these mice were protected from diabetes (72). The phenotype was related to a decrease in autophagosome formation in TECs from mice in which Clec16a was silenced. Interestingly, a general reduction of CD4^+^ T cell activation was observed. The precise mechanism of how Clec16a affects autophagy levels in TECs and, consequently, CD4^+^ T cell selection remains unclear. In addition, it is difficult to link a reduction in autophagosome formation in TECs with an overall hypo responsiveness of CD4^+^ T cells. The authors speculate that the quality of the selected repertoire is different, but no particular auto-antigen specificity was addressed to explain why autoimmunity is dampened. Instead, a global increased negative selection was hypothesized, as shown by a general decrease in CD4SP maturation. How this deficiency will exclusively affect self reactive T cell function without impairing pathogen specific T cell responses, is difficult to understand. Despite that the precise mechanism needs further investigations, the novelty of the study resides in the fact that this is the first demonstration of how CLEC16A can affect autoimmune responses. Indeed, the genetic association of CLEC16A with multiple autoimmune diseases is finally linked to a molecular mechanism impacting autophagy and central tolerance.

**Figure 3:**
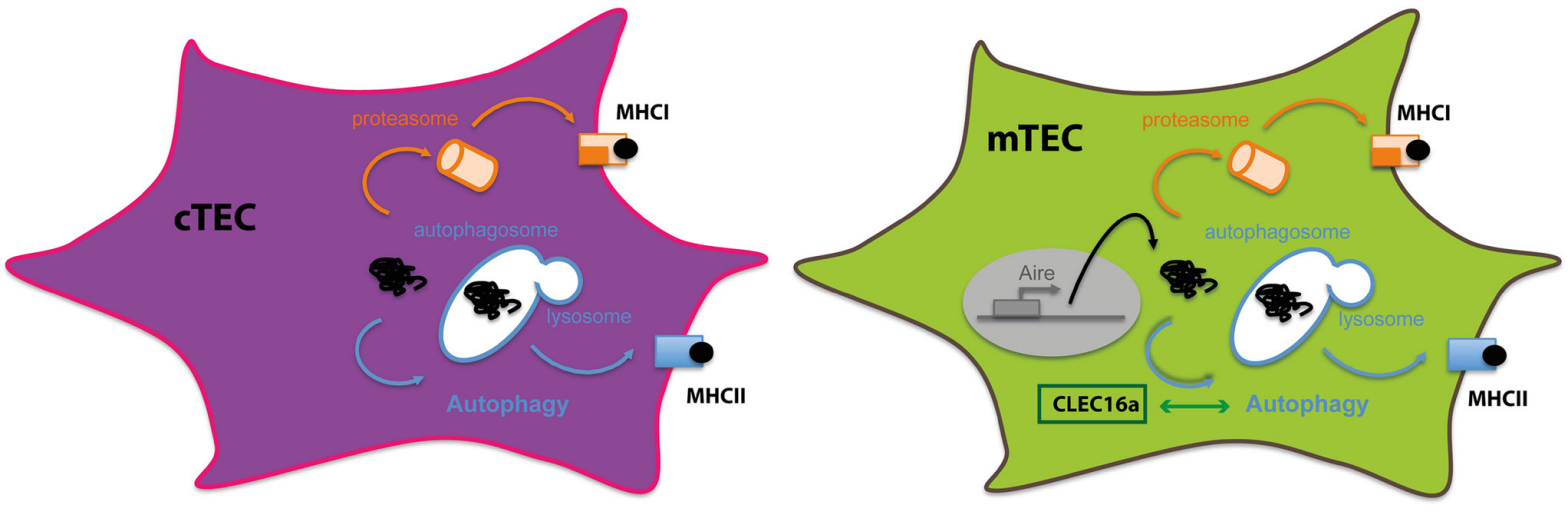
**Autophagy in thymic epithelial cells: thymic epithelial cells are specialized in inducing tolerance**. To sample intracellular- and extracellular-derived antigens, TECs rely on different mechanisms to present antigens via MHC class I or class II molecules. Autophagy in both cTECS and mTECS plays an important role in unconventional cytosolic peripheral self-antigens presentation via MHC class II molecules to establish CD4^+^ T cell tolerance.

Therefore, using non-redundant mechanisms, thymic APCs contribute to efficient CD4^+^ thymocyte differentiation and establishment of CD4^+^ T cell repertoire. Intrinsic features of each subset determine the pathways by which they obtain and process antigens for MHC class II loading. TECs constitute a unique non-hematopoietic cell subset expressing constitutively high levels of MHC class II but exhibiting a poor efficacy in capturing extracellular antigens. With disparities between cTECs and mTECs, macroautophagy has been convincingly demonstrated to participate in the effective loading of intracellular antigens onto MHC class II molecules for the essential process of central tolerance (Figure 3).

## Conclusion

With the advance of the molecular era of autophagy and the identification of ATG genes and pathways, increasing research has demonstrated a prominent role for autophagy in previously unknown biological functions, including adaptive immunity (73). In this regard, autophagy plays an important new role in endogenous antigen processing and presentation of intracellular antigens through MHC class II molecules, with an important effect on CD4^+^ T cell responses. Indeed, the presentation of self-antigens in the thymus via autophagic pathways significantly contributes to shaping the T cell repertoire and to establishing central T cell tolerance.

In addition through enhancing MHC class II presentation of intracellular pathogen-derived antigens, autophagy contributes to efficient CD4^+^ T cell priming and actively shapes adaptive immune responses. Therefore, a better understanding of autophagic functions could be explored to increase the efficiency of vaccines. Moreover, it still remains to be elucidated whether autophagy is also involved in the presentation of self-antigens outside the thymus and if it would, then, play a role in peripheral CD4^+^ T cell tolerance induction and maintenance. Whether activation or suppression of autophagy could have therapeutic benefits in autoimmunity as well as inflammatory disorders requires further clarification.

## Conflict of Interest Statement

The authors declare that the research was conducted in the absence of any commercial or financial relationships that could be construed as a potential conflict of interest.

## Funding

Monique Gannagé is supported by the Institute of Arthritis Research and The de Reuter Foundation. Stéphanie Hugues is supported by the Swiss National Science Foundation (PP00P3_152951), the European Research Council (281365), and the Swiss MS Society.
